# A brief transdiagnostic psychological intervention for Afghan asylum seekers and refugees in Austria: a randomized controlled trial

**DOI:** 10.1186/s13063-019-3839-9

**Published:** 2020-01-09

**Authors:** Matthias Knefel, Viktoria Kantor, Andrew A. Nicholson, Jennifer Schiess-Jokanovic, Dina Weindl, Ingo Schäfer, Brigitte Lueger-Schuster

**Affiliations:** 1 Department of Applied Psychology: Health, Development, Enhancement and Intervention, Faculty of Psychology, University of Vienna, Wächtergasse 1, Vienna, 1010 Austria; 2 Department of Basic Psychological Research and Research Methods, Faculty of Psychology, University of Vienna, Liebiggasse 5, Vienna, 1010 Austria; 3 Department of Psychiatry and Psychotherapy, University Medical Center Hamburg-Eppendorf, Martinistraße 52, Hamburg, 20246 Germany

**Keywords:** Psychotraumatology, Refugees, Psychological intervention, Post-migration living difficulties, Problem Management Plus, Transdiagnostic, Low-resource settings, Cognitive behavioral therapy, Psychotherapy, Complex PTSD, mhGAP

## Abstract

**Background:**

Asylum seekers and refugees are at great risk for developing mental disorders. Afghan refugees are a particularly vulnerable group with a low average education and mental health literacy level. Traumatic experiences and hardship before and during migration are predictive of mental health problems. However, post-migration living difficulties (PMLDs) also account for a large proportion of mental distress in such populations, which, critically, are not sufficiently considered in treatment protocols and research investigations. Indeed, the evidence base for the treatment of refugees and asylum seekers is sparse and limited mainly to trauma-specific treatments, where refugees may likely suffer from other mental health problems such as depression or anxiety.

**Methods/design:**

This trial is the first evaluation of a short-term, transdiagnostic treatment protocol for treatment-seeking Afghan refugees which addresses mental health problems and PMLDs while using an adapted version of the Problem Management Plus (PM+) protocol. Here, we will investigate the efficacy of an intervention manual with a prospective, single-center, randomized, assessor-blind, two-group trial among refugees who are on a waiting list for professional mental health treatment. Furthermore, we will investigate participants’ subjective experiences with the intervention manual via in-depth interviews. One hundred twenty people will be assessed and randomly allocated to either the intervention arm or a treatment-as-usual arm. Clinical psychologists will conduct the treatment, and the sessions will take place with a Dari interpreter. The protocol consists of six 90-min sessions. The primary endpoint is the general symptom distress measure, assessed with the General Health Questionnaire 28 (GHQ-28). Secondary endpoints are the Post-Migration Living Difficulties Checklist (PMLDC), the International Trauma Questionnaire (ITQ), the World Health Organization Quality of Life Questionnaire (WHOQOL-BREF), the Psychological Outcome Profile (PSYCHLOPS), service and health care use (assessed with several items), and the Immigrant Integration Index (IPL-12).

**Discussion:**

This trial may provide substantial evidence for a brief transdiagnostic psychological intervention. Here, we intend to contribute to the treatment of mental health problems among Afghan refugees. The assessment of subjective experience with this treatment manual, as well as the evaluation of its clinical applicability, may optimize treatment acceptance and outcomes across a wide range of mental health problems among refugees.

**Trial registration:**

German Clinical Trials Register (DRKS) registration number: DRKS00016538. Universal Trial Number: U1111-1226-3285. Registered on January 7, 2019. https://www.drks.de/drks_web/setLocale_EN.do

## Background

In the present study, we intend to contribute to the treatment of mental illness among Afghan asylum seekers and refugees. There are currently 2.5 million refugees from Afghanistan, making this group the second largest refugee population in the world [1]. In Austria, people from Afghanistan represented the largest group of asylum seekers from 2001 to 2016 and, since then, the second largest group after Syrian asylum seekers [2]. Indeed, once refugees have arrived in a safe country, many individuals suffer from various mental disorders [3] such as post-traumatic stress disorder (PTSD), depression, and anxiety disorders [4, 5]. Critically, individuals who experience traumatic events are also at greater risk to develop other mental disorders [6]. Of importance, the severity of mental illnesses may be even worse for asylum seekers who do not have an official refugee status [7], and it has been shown that the longer the asylum procedure takes, the higher the risk is for future mental health problems [5]. Research has consistently found that traumatic experiences and hardship, experienced both in the country of origin and during migration, are predictive of mental health problems. Interestingly, post-migration living difficulties (PMLDs) are also predictive of mental health problems [8–11], yet they are seldom addressed in psychological therapies or empirical research investigations [12]. The treatment evidence base for help-seeking refugees and asylum seekers is sparse and limited mainly to trauma-specific treatments [13–16], where in their recent review, Koesters, Barbui, and Purgato surmise: “Except for trauma-related conditions, there is almost no evidence on the efficacy of psychosocial interventions in this population” [12]. Trauma-focused therapies have demonstrated efficacy in reducing symptoms of PTSD, depression, and anxiety, but critically there are a limited number of studies considering other aspects such as daily functioning or quality of life; furthermore, it is not yet clear how to best manage distress by PMLDs [11, 15].

Although high-income countries, such as Austria, have one of the highest densities of mental health professionals, the proportion of trauma specialists is rather low and traumatized people often do not receive timely and appropriate treatment [17, 18]. Specialized treatment facilities are overwhelmed by the large numbers of treatment seekers, resulting in long waiting lists of up to more than a year [19, 20]. Adult survivors of traumatic events show specific barriers to treatment that are related to their trauma symptoms [21]. Additionally, refugees from non-Western countries may have a different conceptualization of mental illness and its treatment [22], where Afghan refugees have a relatively low mental health literacy [23]. Indeed, culturally adapted and evidence-based treatments within stepped or collaborative care systems are needed [24]. Barriers toward treatment acceptance might be further reduced by low-intensity treatments that do not focus on traumatic experiences, facilitating motivation for more intense trauma focused therapy, if needed [25]. On balance, low-intensity treatments in general seem to be effective in reducing mental health problems [﻿27﻿, 28]. Although low-intensity treatments are sometimes delivered by trained lay health workers [﻿26﻿], their effectiveness might be further strengthened if delivered by mental health professionals. Whereas psychotherapy is longer in duration, low-intensity interventions could be provided to a larger number of help-seeking refugees in shorter time and thus could constitute an important first step toward recovery.

Transdiagnostic approaches may be appropriate first-line treatments for help-seeking traumatized individuals; however, there is a lack of evidence for these treatments in refugee populations [﻿28﻿], where factors moderating treatment effectiveness should also be identified [15]. Problem Management Plus (PM+) is a recently developed, low-intensity, transdiagnostic treatment manual for individual settings and was developed by the World Health Organization (WHO) [﻿29﻿, ﻿30﻿]. PM+ was developed to help adults impaired by distress in communities who are exposed to adversity, regardless of whether exposure to adversity has caused their problems [﻿27﻿]. Here, transdiagnostic approaches are based on research suggesting that there are significant commonalities across emotional disorders (e.g., [55]). Furthermore, research has shown that a substantial number of people with mental disorders do not have a single disorder but rather have more than one comorbid conditions [56, 57]. Furthermore, PM+ is a suitable protocol for refugee populations because it was developed as a culturally adaptable and broadly applicable treatment. Transdiagnostic manuals address symptoms of common mental disorders without tailoring the treatment protocol to specific disorders [31]. PM+ was evaluated in studies in Kenya and Pakistan; both research teams were part of the larger PM+ research group. Research in Kenya [26, 32] showed that PM+ was more effective in reducing mental health problems than enhanced usual care (EUC). The main outcome was a change in psychological distress (assessed with the General Health Questionnaire 12, or GHQ-12) from baseline to 3 months post-treatment. Women who received PM+ reported significantly less psychological distress, with a moderate effect relative to EUC. The secondary outcomes (PTSD symptoms and functional impairment) also showed significant reductions but these effects were small relative to EUC. Research in Pakistan [33, 34] investigated PM+ in adult primary care attendees in a conflict-affected area. The treatment arm (PM+) showed significant reduction in the primary outcome, anxiety and depression symptoms (assessed with the Hospital Anxiety and Depression Scale, or HADS), with a moderate to large effect relative to EUC. The secondary outcomes (PTSD symptoms, functional impairment, and symptoms of depressive disorder) also showed significant reductions; these effects were moderate relative to EUC. Here, Khan and colleagues [35] report in their qualitative evaluation that PM+ was considered useful by participants, their families, and lay helpers. Other studies showed less clear results, where the treatment did not significantly reduce psychological distress symptoms in two studies [36, 37], although both of these studies were planned and conducted as pilot trials. However, the trends were in favor of a symptom reduction in the treatment arm in all measures compared with the control group in both studies.

Critically, this is the first study to implement a short-term protocol that will address the needs of adult refugees above and beyond trauma-related symptoms. Furthermore, as part of the present study, we will expand the PM+ manual by including interventions specifically addressing PMLDs.

### Objectives

The aims of this study are to evaluate the efficacy of a short-term transdiagnostic treatment protocol (adapted PM+) to address mental health problems and post-migration living difficulties for treatment-seeking Afghan asylum seekers and refugees who are on a waiting list for professional mental health treatment. This intervention aims to reduce typical symptoms of common mental disorders and associated distress via PMLDs.

We hypothesize that participants in the intervention group will show a significant reduction in general mental distress. Additional aims are to investigate the effectiveness of the intervention in (a) reducing symptoms of PTSD and complex PTSD, (b) alleviating the burden associated with PMLDs, (c) improving refugee’s quality of life, and (d) investigating the participants’ subjective experience and tolerance of the intervention.

## Methods

### Trial design

**Fig. 1:**
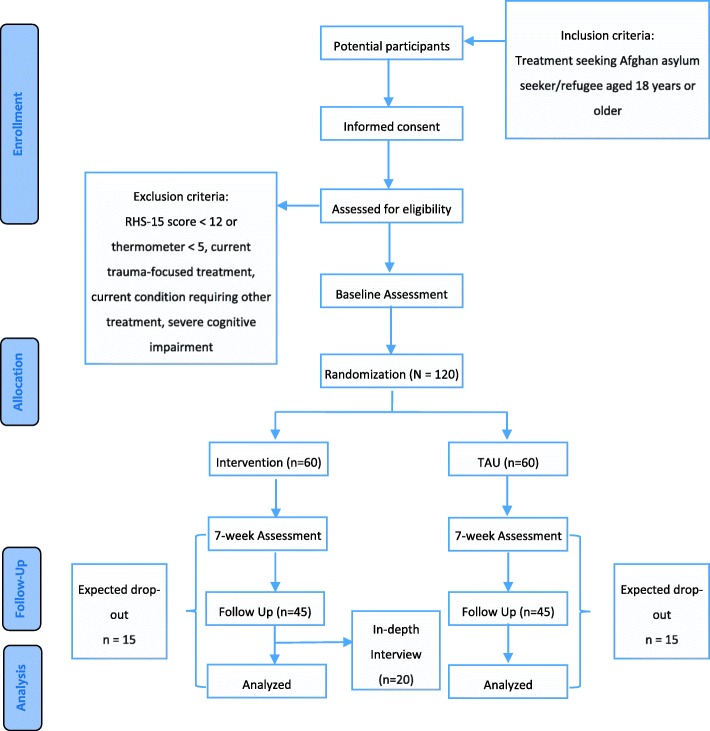
Consolidated Standards of Reporting Trials (CONSORT) flowchart of the study design

This study is designed as a prospective, single-center, assessor-masked, individually randomized, two-group superiority trial. It will compare a six-session transdiagnostic, low-intensity manualized psychological treatment with treatment as usual (TAU). The treatment group will receive TAU and adapted PM+; the control group will receive TAU only. Both groups will be recruited from a professional mental health treatment wait list. The trial design is depicted in the Consolidated Standards of Reporting Trials (CONSORT) flowchart in Fig. 1 [38], the Standard Protocol Items: Recommendations for Interventional Trials (SPIRIT) participant timeline is shown in Table 1, and the SPIRIT checklist [39] is provided as Additional file 1.

**Table 1:**
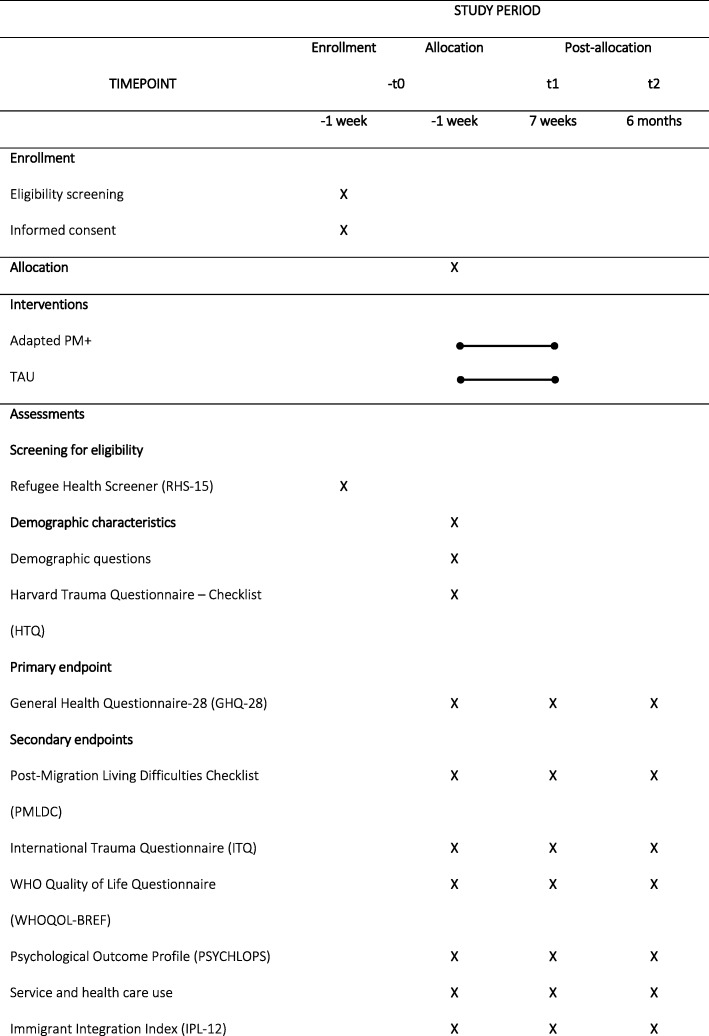
Participant timeline for the Standard Protocol Items: Recommendation for Interventional Trials (SPIRIT)

### Study setting

The trial setting is the Outpatient Unit for Research, Teaching, and Practice, Faculty of Psychology, University of Vienna. All assessments, in-depth interviews, and treatment sessions will take place in a separate room, and only the participant, the psychologist, and the interpreter will be present in the room.

### Participants

The study will include help-seeking, Dari-speaking adult Afghan asylum seekers or refugees who fulfil the following inclusion criteria: (a) being on a wait list for professional mental health treatment in a specialized facility and (b) positive screening on a screening questionnaire for common mental disorders in refugees (a score of 12 or higher on the RHS-15 or a score of 5 or higher on the distress scale of the RHS-15).

Exclusion criteria are (a) presenting with a current condition requiring other treatment (acute suicidality, severe mental disorder such as psychotic disorders or substance dependence), (b) severe cognitive impairment (e.g., moderate to severe intellectual disability or dementia), based on definitions in the Mental Health Gap Action Programme (mhGAP) Intervention Guide [40], or (c) current trauma-focused treatment.

### Intervention

This study uses an adapted version of PM+ in the intervention arm. PM+ [29] is a transdiagnostic, low-intensity psychological treatment for common mental disorders (e.g., depression, anxiety, and stress). It was developed by the WHO as part of its mhGAP [41]. PM+ was developed to help adults impaired by distress in communities that are exposed to adversity, regardless of whether exposure to adversity has caused their problems [29]. The original PM+ manual comprises four core therapeutic strategies that were chosen on the basis of a strong evidence base and following review by international experts [30]. These four core strategies are “Managing Stress”, “Managing Problems”, “Get Going, Keep Doing”, and “Strengthening Social Support”. Our modification of the treatment protocol includes an additional sixth session, focusing on either anger regulation or self-efficacy. This extra session is based on the specific needs of treatment-seeking refugees. It was developed by the research team in the initial phase of the study and includes the results from our preliminary study interviewing experts in the field. Participants will decide during the course of the treatment whether they prefer a session on anger regulation or on self-efficacy, depending on their subjective needs. According to Austrian law, only medical doctors and mental health specialists (clinical psychologists, psychotherapists) have official approval to treat mental disorders. Consequently, the treatment will be implemented by clinical psychologists who have a legal treatment allowance in Austria. Participants have six weekly face-to-face appointments (five from the original protocol and one developed as part of this study); the duration of these sessions is 90 min. The sessions will be facilitated by interpreters. The psychologists will be specifically trained by PM+ master trainers. Regular supervision will guarantee therapist adherence to the protocol. Treatment fidelity will be scored by using the PM+ Helper’s Self-rating Fidelity Checklist. The checklist records delivery of the intervention, psychosocial communication, and safety management skills. The treatment group will receive TAU and adapted PM+.

### Control condition

This study uses a control arm to control for possible spontaneous remission and other uncontrolled factors. The control arm will be assessed at the same time points as the treatment arm but will not receive any treatment by the research team. During the period between pre-assessment and follow-up assessment, participants from this arm will receive TAU in their daily living conditions. We expect that the control arm in this study will receive treatments that are commonly implemented in the Austrian medical settings; this includes any contact with medical and mental health professionals or pharmacological therapy. Receiving psychopharmacological or psychological treatment is considered TAU and does not preclude a person from the control arm.

### Outcome measures

#### Primary outcome

The primary outcome is the general health assessed with the General Health Quenstionnaire 28 (GHQ-28) [42], which is a 28-item self-report questionnaire assessing somatic symptoms, anxiety and insomnia, social dysfunction, and severe depression. The GHQ-28 is widely used in research across different cultural settings and has good psychometric properties [43]. The GHQ-28 will be assessed by investigators blind to treatment allocation at baseline, 7-week follow-up, and 6-month follow-up. We will use the German and Dari versions of the questionnaire.

#### Secondary outcomes

We will also use several secondary endpoints: an adapted Version of the Post-Migration Living Difficulties Checklist (PMLDC) [43], the International Trauma Questionnaire (ITQ) [45], the WHO Quality of Life Questionnaire (WHOQOL-BREF) [44], the Psychological Outcome Profile (PSYCHLOPS) [46], and the Immigrant Integration Index (IPL-12).

The PMLDC is a self-evaluated checklist used to assess the frequency of recent adverse life experiences typical of migration [5]. The checklist was adapted to the study population. The final version includes 26 items measured on a 5-point Likert scale. In addition, the associated degree of distress is assessed on a 10-pont Likert scale. The ITQ is an instrument designed to assess ICD-11 (International Classification of Diseases, 11th ed.) PTSD and complex PTSD. The ITQ includes six items for PTSD, six items for complex PTSD, and two times three items for functional impairment by symptoms of PTSD and complex PTSD [48]. The ITQ has shown good psychometric properties in initial validations [49]. The WHOQOL-BREF is a cross-culturally comparable instrument to assess quality of life. It consists of 26 items measured on a 5-point Likert scale. It is divided into four domains: physical health, psychological health, social relationships, and environment. The PSYCHLOPS promotes a patient-centered definition of therapy outcome and contains questions on problems, function, and well-being. Participants are asked to describe their main problem or problems and how they affect them (function) with five questions. The short version of the IPL-12 [44] is a pragmatic and multidimensional measure of immigrant integration. It consists of 12 items and captures six dimensions of integration: psychological, social, economic, political, linguistic, and navigational. We are using the available Dari and German versions and translating the remaining Questionnaires.

### Additional measures

Traumatic experiences will be assessed with an adapted version of the Trauma Checklist of the Harvard Trauma Questionnaire [51] (Dari and German version). The Service Use Questionnaire (Client Service Receipt Inventory) is used to collect data about the health-care contacts.

### Sample size

Studies using the original PM+ manual so far have found small to large effects compared with TAU at 6 months post-treatment. Assuming an α error probability of 0.05 and a power (1-β error probability) of 0.80, we need a total sample size of 90 persons (45 persons per group) when testing for a medium effect (*f* = 0.3). We chose the more conservative approach and assumed only small effects of the treatment and thus aim to include 90 persons in the analysis. We expect a dropout rate of 25%. Taking this into account, we aim to include a total of 120 participants who will be randomly assigned to either the intervention arm (*n* = 60) or the TAU arm (*n* = 60). A subgroup of the intervention arm (*n* = 20) will be invited for in-depth interviews.

### Recruitment

We will collaborate with non-governmental organizations and outpatient treatment centers in Vienna, Austria, that provide psychological and psychiatric help for treatment-seeking asylum seekers and refugees. These partners have agreed to inform help-seeking Afghan asylum seekers and refugees about our study as part of their initial contact with them giving them our contact information. Participants’ travel costs will be refunded for each assessment and treatment session. Each participant will receive monetary compensation for their involvement (€10; handed out at the 6-month follow-up assessment). As all participants are on a waiting list for professional mental health treatment, it is possible that some might receive treatment during the period of their study participation (i.e., before their 6-month follow-up assessment). As this is considered part of their usual treatment, we do not exclude participants if they receive additional treatment; however, considering the average waiting time of more than a year, we do not expect this in many cases. Information regarding received medical attention and mental health treatment will be gathered during follow-up assessments for both groups.

### Group allocation and blinding

The allocation ratio will be 1:1 with 60 participants per group (total *N* = 120). Participants will be randomly assigned to either group with random allocation software after the baseline assessment. The random numbers will be generated by an assistant who will not be involved in the assessments or in the treatment. The participants will be informed after the baseline assessment. The study will assessor-blind; that is, the psychologist conducting the baseline and follow-up assessments will be masked with regard to the allocated treatment arm (adapted PM+ vs. TAU).

### Data collection and data management

Self-report questionnaires will be offered in Dari and German and will be completed in a face-to-face fully-structured interview with a trained psychologist and an interpreter. All instruments will be delivered in interview format because some participants are expected to be illiterate. The assessment will take about 90 min per time point (baseline, 7-week follow-up, and 6-month follow-up) (i.e., three times 90 min in total for each participant). The in-depth interviews will be conducted by a trained psychologist and an interpreter. They will take about 60 min and will be audio-recorded.

Computer-assisted data assessment will reduce possible data entry errors and increase data quality. All identifying information will be recorded with a participant number to secure participant privacy, and personal identity-related information will be entered into the electronic database containing the assessment information. The key connecting the participant numbers and names (as well as contact and tracking information) will be kept in a separate data file. The electronic files will be saved to an encrypted hard drive with a password lock. Only members of the research team will have access to the respective files. All other papers with identifying information (i.e., signed informed consent forms) will be stored in a separate locked cabinet.

### Statistical analyses

First, we will compare baseline scores between the treatment group and TAU group on all primary and secondary outcome measurements, using chi-squared tests and two-sample *t* tests, for categorical and continuous variables, respectively. In order to estimate significant changes over time as a result of the intervention for all primary and secondary outcome measures, we will employ hierarchical linear modelling (HLM). Analyses will be based on an intent-to-treat population, where critically HLM allows the number of observations to vary between participants and effectively handles missing data by calculating estimates of trajectories using maximum likelihood estimation. Time (linear and quadratic), treatment condition, and their interaction will be included in the models, where we will also examine how covariate factors such as age, biological sex, education level, and asylum status explain variance within the data. Here, similar models will be employed for the primary and secondary outcome measures, and we will conservatively correct for multiple comparisons where appropriate. Specifically, the level 1 model will characterize within-patient change over time, and the level 2 model will predict variation of within-patient change over time and between-patient variables.

We will also examine whether treatment success can be predicted from baseline clinical measures and individual differences using machine learning methods, including non-linear random forest regressions and linear ordinary least squares model with lasso regularization (OLS-LASSO).

In regard to qualitative data, interviews of the sub-sample will be transcribed verbatim and will be analyzed using thematic analysis [45]. The thematic analysis approach is a method for identifying, analyzing, and reporting themes within data. The in-depth interviews will be analyzed independently by two researchers, and inter-rater reliability will be calculated. The results from the interviews following the online training tool will be analyzed with the same methodological approach.

### Data monitoring

All serious adverse events (SAEs) that are reported spontaneously by the participants or observed by the investigators during the trial will be recorded by the research team and will be reported to the independent advisory board [46]. The chair of the advisory board will review SAEs within 72 h, and the research team will review all AEs regularly and, where necessary, respond appropriately. The consent process includes informing participants about whom they can contact if they experience adverse reactions (i.e., Kriseninterventionszentrum Wien [Crisis intervention center Vienna]). Depending on the nature of the adverse event, follow-up may require additional tests or medical procedures as indicated and/or referral to a general physician or medical specialist. All adverse events will be followed until specialist care (including referrals, additional tests, or medical procedures) is in place for the client or until a stable situation has been reached.

## Discussion

There currently exists an urgent need for care among Afghan asylum seekers and refugees in order to address mounting mental health problems and post-migration stressors among these populations. This clinical trial is expected to provide evidence for the efficacy of an adapted short-term treatment protocol (adapted PM+). This evaluation is necessary for implementing early and widespread treatment methods, where low-intensity treatments might effectively reduce symptom burden and thus prevent chronification of symptoms when administered early. Transdiagnostic approaches may be appropriate first-line treatments for help-seeking traumatized individuals, and mental health professionals without training in trauma therapy could provide effective initial treatment options for traumatized refugees. A few limitations of the study exist regarding the TAU and the participant characteristics. The first limitation is that we are not able to employ active control conditions. Second, because participant recruitment would be hindered, we will not be able to limit changes to pharmacotherapy during the course of this study. Third, we can only take account of participants being on a waiting list for professional mental health treatment in a specialized facility, which limits the generalizability of the results. Despite these limitations, this study is expected to provide essential information regarding mental health care for Afghan asylum seekers and refugees.

### Trial status

Protocol version number 1.0 (Aug. 12, 2019). This study has been recruiting participants since June 2019; recruitment is planned to be completed in September 2020.

## Supplementary information

**Additional file 1.** SPIRIT (Standard Protocol Items: Recommendations for Interventional Trials) checklist.

## Data Availability

Not applicable.

## Abbreviations

EUC: Enhanced usual care
GHQ-28: General Health Questionnaire-28
HLM: Hierarchical linear modeling
IPL-12: Immigrant Integration Index
ITQ: International Trauma Questionnaire
mhGAP: Mental Health Gap Action Programme
PM+: Problem Management Plus
PMLD: Post-migration living difficulty
PMLDC: Post-Migration Living Difficulties Checklist
PSYCHLOPS: Psychological Outcome Profile
PTSD: Post-traumatic stress disorder
RHS-15: Refugee Health Screener
SAE: Serious adverse event
SPIRIT: Standard Protocol Items: Recommendations for Interventional Trials
TAU: Treatment as usual
WHO: World Health Organization
WHOQOL-BREF: World Health Organization Quality of Life Questionnaire
